# Effect of a specific composition of short- and medium-chain fatty acid 1-Monoglycerides on growth performances and gut microbiota of gilthead sea bream (*Sparus aurata*)

**DOI:** 10.7717/peerj.5355

**Published:** 2018-07-31

**Authors:** Simona Rimoldi, Emi Gliozheni, Chiara Ascione, Elisabetta Gini, Genciana Terova

**Affiliations:** 1 Department of Biotechnology and Life Sciences, University of Insubria, Varese, Italy; 2 Inter-University Centre for Research in Protein Biotechnologies, “The Protein Factory”, Polytechnic University of Milan and University of Insubria, Varese, Italy

**Keywords:** Next-generation sequencing, Metagenomics, Gut microbiome, Feed additive, Aquaculture, 1-Monoglycerides, SILOhealth 108Z, 16S rRNA gene

## Abstract

In aquaculture research, one important aim of gut microbiota studies is to provide the scientific basis for developing effective strategies to manipulate gut microbial communities through the diet, promoting fish health and improving productivity. Currently, there is an increasing commercial and research interest towards the use of organic acids in aquafeeds, due to several beneficial effects they have on growth performance and intestinal tract’s health of farmed fish. Among organic acids, monoglycerides of short-chain fatty acids (SCFAs) and medium-chain fatty acids (MCFAs) have attracted particular research attention also for their bacteriostatic and bactericidal properties. Accordingly, the present study aimed to evaluate the potential beneficial effects of SCFA and MCFA monoglycerides, used as a feed additive, on fish growth performance, and intestinal microbiota composition. For this purpose, a specific combination of short- and medium-chain 1-monoglycerides (SILOhealth 108Z) was tested in 600 juvenile gilthead sea bream (Sparus aurata) of about 60 g mean initial weight that were fed for 90 days with plant-based diets. Two isoproteic and isolipidic diets were formulated. The control fish group received a plant-based diet, whereas the other group received the same control feed, but supplemented with 0.5% of SILOhealth 108Z. The Illumina MiSeq platform for high-throughput amplicon sequencing of 16S rRNA gene and QIIME pipeline were used to analyse and characterize the whole microbiome associated both to feeds and S. aurata intestine. The number of reads taxonomically classified according to the Greengenes database was 394,611. We identified 259 OTUs at 97% identity in sea bream fecal samples; 90 OTUs constituted the core gut microbiota. Firmicutes, Proteobacteria and Actinobacteria represented the dominant phyla in both experimental groups. Among them, relative abundance of Firmicutes and Proteobacteria were positively and negatively affected by dietary SCFA monoglycerides supplementation, respectively. In summary, our findings clearly indicated that SILOhealth 108Z positively modulated the fish intestinal microbiota by increasing the number of beneficial lactic acid bacteria, namely, Lactobacillus, and reducing Gammaproteobacteria, which include several potential pathogenic bacteria. The specific composition of 1-monoglycerides of short- and medium-chain fatty acids contained in SILOhealth 108Z could thus have a great potential as a feed additive in aquaculture.

## Introduction

Aquaculture, with an average annual rate of 8 percent, is probably the fastest-growing food-producing sector in the world. It provides nearly 50 percent of the seafood consumed by humans (FAO, 2014) and the World Bank projects that aquaculture will increase to provide 2/3 of the world’s fish in 2030. Currently, about 68% and 88% of the demand for fishmeal (FM) and fish oil (FO), respectively, comes from aquaculture (Naylor et al., 2009). However, with most wild fish capture fisheries at or above maximum sustainable yield, aquaculture can no longer rely on oceanic resources for the manufacturing of aquafeeds and such feed options are simply not sustainable (Naylor et al., 2000). To defray rising costs and avert ecological harm, commercial feed producers and fish farmers have made substantial efforts to reduce the proportion of FM and FO in aquaculture feed, by replacing ground-up forage fish with terrestrial plants (Tacon & Metian, 2008; Gatlin et al., 2007). However, the use of vegetable feedstuff in aquafeed production has several drawbacks that are related to the low level of indispensable amino acids (in particular lysine and methionine) and to the presence of a wide variety of anti-nutritional factors that could damage the intestine, thus reducing nutrient absorption, and consequently, affecting fish growth and resistance to stress and diseases (Zhang et al., 2013; Penn et al., 2011; Santigosa et al., 2011; Francis, Makkar & Becker, 2001).

For this reason, nutritionists and feed manufacturers are investing great effort to find feed additives that could prevent or alleviate the adverse effects at the gut level of plant-based ingredients that are actually used in fish diet formulations.

Here, the most promising feed additives seem to be organic acids that are compounds with acidic properties associated with their carboxyl group (−COOH) (Lim et al., 2015).

Among them, short- and medium-chain fatty acids (SCFAs and MCFAs) are known to play a central role as energy-source for enterocytes. SCFAs are fatty acids with aliphatic tails of one to six carbon atoms, the most common being acetic (C2), propionic (C3), and butyric (C4) acid, whereas MCFA comprise fatty acids with seven to 12 carbon atoms. SCFAs are produced within the intestinal lumen by bacterial fermentation of undigested dietary carbohydrates and fibers (cellulose, hemicellulose, pectin). Contrariwise, MCFAs mainly arise from dietary triglycerides and natural sources of MCFAs are generally coconut oil, palm kernel oil, and milk. The use of SCFAs as additive in aquafeeds and their impact on fish growth, nutrient utilization, and disease resistance were recently reviewed (Ng & Koh, 2017). Among SCFAs, butyric acid has received particular attention for its various well-documented beneficial effects on the health of intestinal tract and peripheral tissues in human and farmed animals, including fish (Guilloteau et al., 2010; Mátis et al., 2013; Robles et al., 2013; Liu et al., 2014)*.* Butyrate represents a major energy source for enterocytes and is involved in maintaining gut mucosal health, playing a central role in enhancing epithelial cell proliferation and differentiation and in improving the intestinal absorption (Gálfi & Neogrády, 2001; Wong et al., 2006; Canani et al., 2011). Butyrate has anti-inflammatory properties and the potential to stimulate the immune system, too (Vinolo et al., 2011; Hamer et al., 2008; Terova et al., 2016; Rimoldi et al., 2016; Tian et al., 2017). However, the data on the effect of butyric acid and its salts (sodium butyrate) on the growth performance of cultured fish and crustaceans are still controversial. In juvenile common carp (*Cyprinus carpio*) (Liu et al., 2014), and Pacific white shrimp (*Litopenaeus vannamei*) (Da Silva et al., 2016), butyrate supplementation positively affected the growth performance. On the other hand, a dietary supplementation of a mixture of SCFAs, containing butyrate, did not significantly improve growth rate or feed utilization in Atlantic salmon (*Salmo salar*), rainbow trout (*Oncorhynchus mykiss*), and European sea bass (Bjerkeng, Storebakken & Wathne, 1999; Gao et al., 2011; Terova et al., 2016). Recently, Simó-Mirabet et al. (2017) reported that sodium salt of coconut fatty acid distillate, particularly rich in lauric acid (C12), increased feed intake, improved gut development and nutrient absorption, thus enhancing growth rate of gilthead sea bream (*Sparus aurata*). Moreover, MCFAs have been suggested to have a role in immunological response regulation (Wang et al., 2006). Organic acids, their salts or combinations thereof, are commonly known as acidifiers and are used as storage preservatives in terrestrial livestock feeds as well as in aquafeeds (Ng & Koh, 2017). Due to their capacity to reduce pH, they inhibit microbial growth and diminish a possible contamination of feed by pathogenic organisms such as *Salmonella* and *Escherichia coli* (Lückstädt, 2008; Van Immerseel et al., 2003; Van Immerseel et al., 2004; Skřivanová et al., 2009)*.* The mechanism of action of SCFAs and MCFAs differs from that of antibiotics. Salsali, Parker & Sattar (2008) firstly proposed that SCFAs and MCFAs bacteriostatic and bactericidal activities could be due to the ability of the undissociated form of the acid to penetrate the bacterial cell wall and, once inside, to dissociate releasing protons, thereby lowering the cytoplasmic pH. Consequently, the bacterium must redirect its energy towards the efflux of the excess protons, thus exhausting cell metabolism and leading to lower bacterial cell growth and even to cell death (Salsali, Parker & Sattar, 2008; Hismiogullari et al., 2008; Ng & Koh, 2017). In the digestive tract, organic acids cause a pH reduction in the intestine via the delivery of H^+^ ions (Lim et al., 2015). Actually, in fish, dietary administration of acidifiers inhibits overgrowth of pH-sensitive pathogenic bacteria favouring the growth of beneficial intestinal flora (Zhou et al., 2009; Hoseinifar, Sun & Caipang, 2017; Abu Elala & Ragaa, 2015; Ringøet al., 2016; Da Silva et al., 2013; Da Silva et al., 2016; Anuta et al., 2011; De Schryver et al., 2010; Liu et al., 2014; Piazzon et al., 2017). Indeed, although the bacteriostatic activity of organic acids is preserved at the intestinal level, their bactericidal efficacy is limited because of the intestinal pH. Being weak acids with modest pKas of approximately 3.6 to 4.7, the majority of organic acids at neutral or slightly alkaline pH, are present as anions rather than as undissociated forms (free acids) that are assumed to penetrate the lipid membrane, destroying the bacterial cell (Yoon et al., 2018).

Dietary free organic acids and their salts have also the disadvantage to be easily absorbed by the upper digestive tract, thus limiting their delivery to the desired target, i.e., lower intestinal tract, where they exert the aforementioned beneficial actions.

On the contrary, monoglycerides, which are esters formed by glycerol and one molecule of fatty acid, have no such drawbacks. The great advantage of monoglycerides is that organic acid is released from the glycerol backbone only under the action of intestinal lipases. This means that SCFA or MCFA remains protected from absorption in the upper gastrointestinal tract and could reach the final portion of intestine, where it would exert its major functions (Sampugna et al., 1967; Namkung et al., 2011). Moreover, monoglycerides possess a more effective antimicrobial activity than the corresponding free fatty acids, since their efficacy is independent from environmental pH (Bergsson et al., 2001; Sun, O’Connor & Roberton, 2003; Thormar, Hilmarsson & Bergsson, 2006). Due to their amphipathic properties, monoglycerides show a membrane-lytic action, which leads to bacterial membrane destabilization and pore formation. Membrane-destabilizing activity causes increased cell permeability and cell lysis, leading to inhibition of growth and cell death (Yoon et al., 2018). MCFA monoglycerides are able to penetrate also the peptidoglycan layer of Gram-positive bacteria’s cell wall (Bergsson et al., 2001).

Up to date, antimicrobial and growth-promoting action of monoglycerides have been widely investigated in poultry (Bedford & Gong, 2018; Yang et al., 2018; Jahanian & Golshadi, 2015; Leeson et al., 2005), whereas in fish their effects have been poorly explored. Accordingly, the present study aimed to evaluate the potential beneficial effects of dietary SCFA and MCFA monoglycerides on fish growth performances and intestinal microbiota composition. For this purpose, a specific synergic combination of 1-monoglycerides of short- and medium-chain fatty acids (SILOhealth 108Z), commercially available from SILO SpA, Florence, Italy (http://www.silohealth.com/), was tested in juvenile gilthead sea bream (*Sparus aurata*) fed a plant-based diet. The Illumina MiSeq platform for high-throughput sequencing of 16S rRNA gene was utilized to analyse and characterize the whole gut microbiome of gilthead sea bream.

## Materials and Methods

### Ethics statement

This study was carried out in strict accordance with the recommendations in the Guide for the Care and Use of Laboratory Animals of the indoor experimental facility of Civita Ittica (Civitavecchia, Italy), and in accordance with EU Directive 2010/63/E U for animal experiments. The Committee on the Ethics of Animal Experiments of the same experimental facility approved all of the study protocols (approval n. 120/2008-A of 03/09/2008 (Art.12 of D.Lgs.116/92)). Fish handling was performed under tricaine methanesulfonate (MS222) anesthesia, and all effort was made to minimize discomfort, stress, and pain to the fish.

### Experimental diets

The two experimental diets were formulated and manufactured by VRM S.r.l. Naturalleva (Verona, Italy). Feeds were prepared using small-scale machinery for mixing ingredients and preparing pellets of 3.0 mm in diameter. The formulation and proximate composition of diets are shown in Tables 1 and 2. The diets were isoenergetic (17.5 MJ kg^−1^), *isoproteic* (50%), and *isolipidi* c (16%), fully satisfying the gilthead sea bream nutritional demands (Table 2). The control group (CTRL) received a commercial plant-based diet; the treated group (Sh108) received the same control feed but it was supplemented with 0.5% of SILOhealth 108Z commercially available from SILO SpA, Florence, Italy (http://www.silohealth.com/). SILOhealth 108Z is composed of a specific combination of 1-monoglycerides of short- and medium-chain fatty acids (from C3 to C12), in which 1-monobutyrin represents 65% of total blend (Table 3).

**Table 1:** Formulation (g kg^−1^ diet) of experimental diets.

Ingredient	CTRL	Sh108
Fish meal	280.0	280.0
Corn gluten	220.0	220.0
Guar germ meal	132.0	132.0
Soybean seed meal	120.0	120.0
Wheat middlings	120.0	120.0
Fish oil (94%)	64.5	62.4
Rapeseed oil	44.3	41.4
DL-methionine	4.5	4.5
Lysine hydrochloride	2.7	2.7
Taurine	4.5	4.5
Vitamin C (stay-C 35)	0.6	0.6
Vitamin and mineral premix^a^	7.0	7.0
SILOhealth108	–	5.0

**Notes.**

a Vitamin and mineral premix (quantities in 1 kg of mix): Vitamin A, 4,000,000 IU; Vitamin D3, 800,000 IU; Vitamin C, 25,000 mg; Vitamin E, 15,000 mg; Inositol, 15,000 mg; Niacin, 12,000 mg; Choline chloride, 6,000 mg; Calcium Pantothenate, 3,000 mg; Vitamin B1, 2,000 mg; Vitamin B3, 2,000  mg; Vitamin B6, 1,800 mg; Biotin, 100 mg; Manganese, 9,000 mg; Zinc, 8,000 mg; Iron, 7,000 mg; Copper, 1,400 mg; Cobalt, 160 mg; Iodine 120 mg; Anticaking & Antioxidant + carrier, making up to 1,000 g.

**Table 2:** Proximate composition (g kg^−1^ diet) of the experimental diets.

	DIET	
	CTRL	Sh108
Moisture	42.1	42.1
Crude protein	500.0	500.0
Crude lipids	160.0	160.0
Crude fibre	19.6	19.6
NFE	213.3	213.3
Ash	65.0	65.0
DP	403.9	403.9
DE (MJ kg ^−1^)	17.5	17.5
DP/DE (g MJ ^−1^)	22.9	23.0
EPA	12.3	11.8
DHA	8.2	7.8
*n* − 3∕*n* − 6	1.3	1.3
DHA/EPA	0.6	0.6

**Notes.**

NFE Nitrogen-free extract DP digestible protein DE digestible energy EPA Eicosapentaenoic acid DHA Docosahexaenoic acid *n* − 3 omega-3 fatty acids *n* − 6 omega-6 fatty acids

**Table 3:** Fatty acid composition (%) of SILOhealth 108Z.

	Fatty acid	Quantity (%)
C3:0	Propionic acid	20
C4:0	Butyric acid	65
C6:0, C7:0, C8:0, C9:0, C12	Blend of caproic, heptanoic, caprylic, lauric acid	15

### Fish and feeding trial

Six hundred juvenile gilthead sea bream of about 60 g mean initial body weight (Table 4) were randomly distributed into six fiberglass tanks of 2 m^3^ each (100 fish/tank) at the indoor experimental facility of Civita Ittica (Civitavecchia, Italy). The tanks were supplied with filtered sea water (salinity of 37 mg/l) at a temperature and average dissolved oxygen level of 21.2 ± 1.4°C and 11.7 ± 0.6 mg/l, respectively. Fish were kept under a 12:12 h light:dark photoperiod regimen. Feeding rate was restricted to 2.0% of biomass during the feeding experiment based on four-weekly fish weight measurements. During the experiment that lasted 90 days, fish in triplicate groups (three tanks/diet) were fed with their respective diet twice a day (7:00 am and 4:00 pm) for 6 days per week, except Sunday. Feed consumption (g) in each tank was estimated from the difference between feed delivered into the tank and uneaten feed. Uneaten feed was collected from the bottom of the tank one hour after each meal by siphoning, dried at 70 °C and then weighed. Fish mortality was checked and recorded every day. At the end of the feeding trial, all fish in the tank were individually weighed and measured for their length. Specific growth rate (SGR), relative growth rate (RGR), and biological and economic feed conversion ratio (bFCR and eFCR, respectively) values were calculated. The bFCR is the net amount of feed used to produce one kg of fish, whereas the eFCR considers all the feed used, meaning that the effects of feed losses and mortalities are included (Robb & Crampton, 2013).

**Table 4:** Growth and feed efficiency indices. Final mean body weight, specific growth rate (SGR), relative growth rate (RGR), biological feed conversion ratio (bFCR), and economic feed conversion ratio (eFCR) values of sea bream fed with two experimental diets (CTRL and Sh108). The weight data represent the mean value ± SD (*n* = 300 fish/per diet). SGR, RGR, bFCR, and eFCR were tank-based determined (*n* = 3) and reported as mean ± SD. Different letters indicate statistically significant differences between groups (Student’s *t*-test, *P* < 0.05).

Diet	Initial weight	Final weight	SGR (% day^−1^)	RGR (%)	bFCR	eFCR
CTRL	60.56 ± 1.44	126.84 ± 1.90	0.75 ± 0.01	109.49 ± 2.49	1.53 ± 0.05	1.55 ± 0.05^a^
Sh108	60.50 ± 0.70	129.39 ± 1.12	0.77 ± 0.01	113.88 ± 3.27	1.47 ± 0.01	1.48 ± 0.01^b^

The each ratio values were calculated using the following formulas:

\begin{eqnarray*}\text{bFCR}& =\text{Total feed} /(\text{Final weight} \left( {\mathrm{W}}_{\mathrm{t}} \right) +\text{mass mortality})-\text{Initial weight}  \left( {\mathrm{W}}_{0} \right) \end{eqnarray*}

\begin{eqnarray*}\text{eFCR}& =\text{Total feed} /(\text{Final weight} \left( {\mathrm{W}}_{\mathrm{t}} \right) -\text{Initial weight}  \left( {\mathrm{W}}_{0} \right) ) \end{eqnarray*}

\begin{eqnarray*}\mathrm{SGR}& =100 \times   \left( \ln \nolimits {\mathrm{W}}_{\mathrm{t}}/\ln \nolimits {\mathrm{W}}_{0} \right) /\text{Days} \end{eqnarray*}

\begin{eqnarray*}\text{RGR}& =100 \times \left( {\mathrm{W}}_{\mathrm{t}} \right. - \left. {\mathrm{W}}_{0} \right) /{\mathrm{W}}_{0}. \end{eqnarray*}

The day of fecal sampling, fish were fed at 6:00 am and after 6 h from the last meal, six fish/diet (2 fish/tank) were randomly collected and euthanized with an overdose (320 mg/L at 22 °C) of anesthetic (tricaine-methasulfonate MS-222). To avoid gut content contamination by the body surface microflora during dissection, external abdominal surface of each fish was wiped thoroughly with a sterile 70°alcohol moistened cotton with an area of 10 cm^2^. Then, with the aid of sterile scissors and forceps, the entire intestine (excluding pyloric ceca) was exposed from the ventral side and aseptically removed. The fecal content was obtained by squeezing out and scrapping the intestinal mucosa with a sterile spatula, in order to collect both, the digesta- and the mucosa-associated microbiota. The fecal samples were immediately frozen in dry ice and stored at minus 80 °C until the metagenomics analysis.

### Microbial DNA extraction

Two hundred and fifty mg of intestinal content from each fish (12 × 250 mg samples in total) and 200 mg of each dietary pellet (2 × 200 mg samples in total) were processed for DNA extraction using DNeasy PowerSoil Kit (Qiagen, Milan, Italy). The bacterial cells were disrupted via high-speed shaking in plastic tubes with stainless steel beads (TissueLyser II, Qiagen, Milan, Italy) for 2 min at 25 Hz. Total DNA was then extracted according to the manufacturer’s instructions. A sample with only lysis buffer was processed in parallel to the biological samples as a negative control to check if external DNA contamination was introduced during the extraction procedure. Bacterial DNA concentration was measured spectrophotometrically by using NanoDrop™ 2000 Spectrophotometer (Thermo Fisher Scientific, Monza, Italy) and then stored at −20 °C until further processing.

### 16S rRNA gene library preparation and sequencing

The 16S ribosomal RNA gene library was prepared according to the Illumina protocol “16S Metagenomic Sequencing Library Preparation” (#15044223 rev.B). PCR amplifications of the V3-V4 region of the 16S rRNA gene were carried out in 25-µl reactions containing bacterial DNA (500 ng), buffer (10X), dNTPs (0.2 mM), MgSO_4_ (1.5 mM), Platinum^^®^^ Taq DNA Polymerase High Fidelity (1U) (Thermo Fisher Scientific, Monza, Italy), forward primer (5′-CCTACGGGNBGCASCAG-3′), and reverse primer (5′-GACTACNVGGGTATCTAATCC-3′) (400 nM each). The universal primers used were selected by Takahashi et al. (2014) and were designed with Illumina adapters at their 5′ end. All the procedure for 16S rRNA gene library preparation and sequencing is described in detail in Rimoldi et al. (2018). However, briefly, PCR cycling conditions for 16S rRNA gene amplification were 94 °C for 1 min, 30 cycles of 94 °C for 30 s, 55 °C for 1 min, and 68 °C for 1.30 min, with a final extension step at 68 °C for 10 min. The resulting size of 16S rRNA gene amplicons was about 550 bp. Dual indices and Illumina sequencing adapters (P5 and P7) were then attached to the amplicons using Nextera XT Index Kit (Illumina, San Diego, CA, USA), according to manufacturer’s instructions, to produce the final libraries. Final libraries were quantified by quantitative PCR (qPCR) using KAPA Library Quantification Kits for Illumina^®^ platforms (Kapa Biosystems Ltd., Dorset, UK) and a set of six diluted DNA standards to generate a standard curve. Final libraries were pooled in equimolar amounts, denatured and diluted to 6 pM. Before loading onto the MiSeq flow cell, 15% of the PhiX control library was combined with the amplicon library. Sequencing was performed on an Illumina MiSeq platform using v3 reagent and a 2 × 300 bp paired end protocol, according to the manufacturer’s instructions (Illumina, San Diego, CA, USA).

### Sequencing raw data analysis

Raw sequences were processed using the open-source bioinformatics pipeline QIIME v1.9.1 (Caporaso et al., 2010) by BMR Genomics NGS service (Padova, Italy). Sequences were trimmed using Trimmomatic v0.32. Only reads above 36 nucleotides in length were included in the downstream analysis. The remaining sequences were grouped by diet according to their barcodes. For original amplicon reconstruction, overlapping R1 and R2 paired reads were joined using FLASH v1.2.11 software (http://sourceforge.net/projects/flashpage) and filtered for base quality (Q > 30). Amplicons were dereplicated, sorted, and clustered at ≥ 97% identity. Amplicon clusters (Operational Taxonomic Units, OTUs) were then identified against reference QIIME-formatted Greengenes database v.13.8 (http://greengenes.lbl.gov) by using QIIME script ‘pick_closed_reference_otus.py’ and only the OTUs that represented at least 0.005% of total reads were kept. The taxonomical classification was performed down to species level. To determine the abundance of each bacterial taxon, OTUs obtained from each sample were binned according to their consensus sequences, and the final OTU-table output files, in txt and biom format, were created using ‘summarize_taxa_through_plots.py’ custom script. OTUs assigned to the phylum *Cyanobacteria* (class *Chloroplast*) were removed from the analysis as potential plant contaminants, as described in Rimoldi et al. (2018). Reads of mitochondrial or eukaryotic origin were also excluded.

Alpha and beta diversity statistics were performed as described in Rimoldi et al. (2018). Alpha diversity metrics were calculated based on a rarefied OTU table using ‘observed species’, ‘Chao1 index’ (species richness estimator), ‘Shannon’s diversity index’, ‘Good’s coverage’, and ‘PD whole tree’. OTUs diversity among sample communities (beta diversity) was assessed by applying weighted (presence/absence/abundance matrix) and unweighted (presence/absence matrix) UniFrac distance matrices (Lozupone & Knight, 2005; Lozupone et al., 2007). The distance matrices were visualized by principal coordinate analysis (PCoA) three-dimensional plots.

The common core microbiome (OTUs shared, regardless of the diet, and found in at least five out of the six samples per dietary group) was identified using the ‘compute_core_microbiome.py’ script. The Venn diagrams representing the results of the core microbiota were drawn using the web tool http://bioinformatics.psb.ugent.be/webtools/Venn/.

### Statistics

All data were presented as means ± standard deviation. The number of reads across samples was normalized by sample size and the relative abundance (%) of each taxon was calculated. Only those taxa with an overall abundance of more than 1% (up to order) and more than 0.5% at family and genus level were considered for statistical analysis. Before being statistically analysed, the resulting microbial profiles were calculated as the angular transformation (arcsine of the square root). All data were tested for normality and homogeneity of variances by Shapiro–Wilk’s and Levene’s test, respectively. Differences between two groups were analysed by unpaired Student’s *t*-test or non-parametric Mann–Whitney U test, depending if the data were or not normal distributed. Welch’s *t*-test was used instead of Student’s *t*-test when variances were unequal between groups. Statistical significance was set at *P* < 0.05. Correction of multiple testing was done using Benjamini–Hochberg False Discovery Rate (FDR) method with a false discovery rate (*Q*) set to 0.20. All analyses were performed using Past3 software (Hammer, Harper & Ryan, 2001). To verify the significance of differences in the beta diversity of bacterial communities, analysis of similarities (ANOSIM), and permutational multivariate analysis of variance (adonis function) were performed with 999 permutations. Both tests were accomplished using QIIME script ‘compare_categories.py’.

## Results

### Fish growth performance and feeding conversion

During the 90 days of the feeding trial, the mortality rate was lower than 1%. Specifically, two fish of CTRL and four fish of Sh108 group died during the first week of feeding trial, with no further mortalities recorded for the rest of the test. Fish growth performance indexes such as SGR, and RGR did not reveal any significant differences between control and SILOhealth 108Z-supplemented dietary groups, meaning that all fish grew efficiently, regardless of the fatty acid monoglycerides supplementation. At the end of the feeding trial, all fish doubled their body mass reaching a final mean body weight of 126.84 ± 1.90 g, and 129.39 ± 1.12 g in CTRL and Sh108 group, respectively. On the contrary, economic FCR differed between two groups, resulting lower in fish fed diet Sh108 (Table 4).

### Characterization of microbial communities of the diets

**Figure 1:**
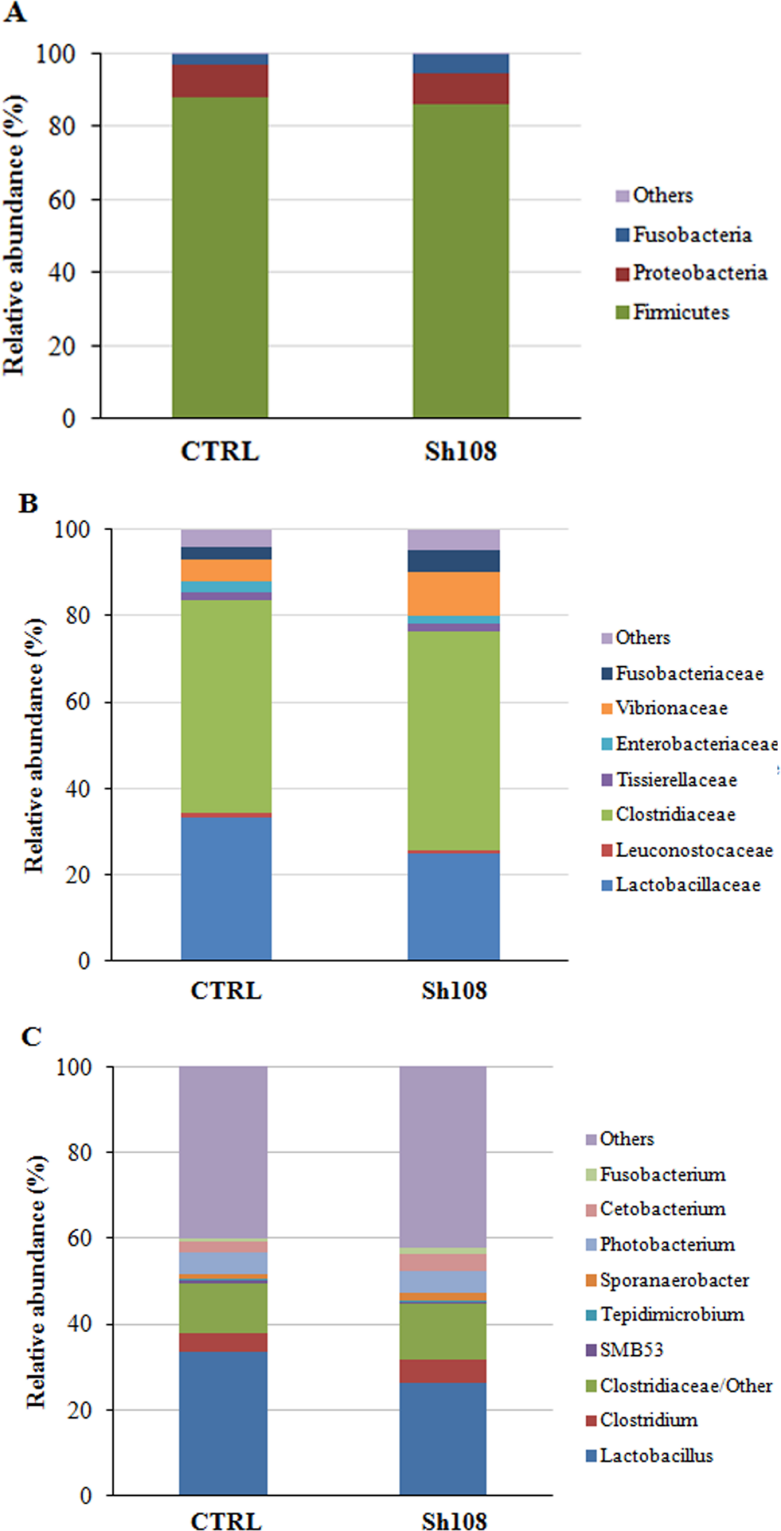
Bacterial relative abundance (%) in the feeds. The amount (%) of the most prevalent bacteria in CTRL and Sh108 feeds at (A) phylum; (B) family, and (C) genus level. Only bacteria with an overall abundance of ≥ 1% (at genus level) and ≥ 0.5% (at family and genus level), were reported. Bacteria with lower abundance were pooled and indicated as “Others”.

Bacterial communities associated to feeds were analysed using the QIIME pipeline, which revealed that the two microbial profiles were qualitatively and quantitatively equivalent. After filtering for quality, trimming length, and generating consensus lineages, the number of reads taxonomically classified according to the Greengenes database was 47,791 and 44,483 for CTRL and Sh108 diet, respectively. The total number of OTUs at 97% identity found in CTRL and Sh108 feed samples amounted to 193 and 188, respectively. The overall amount of reads of eukaryotic origin was around 70%. The microbial profiles of feed samples at the phylum, family, and genus taxonomic level are reported in Figs. 1A–1C. The most abundant bacterial taxa (relative abundance >1%) were mainly comprised of 3 phyla, four classes, six orders, seven families, eight genera, and eight species (Figs. 1A–1C; Dataset S1).

**Figure 2:**
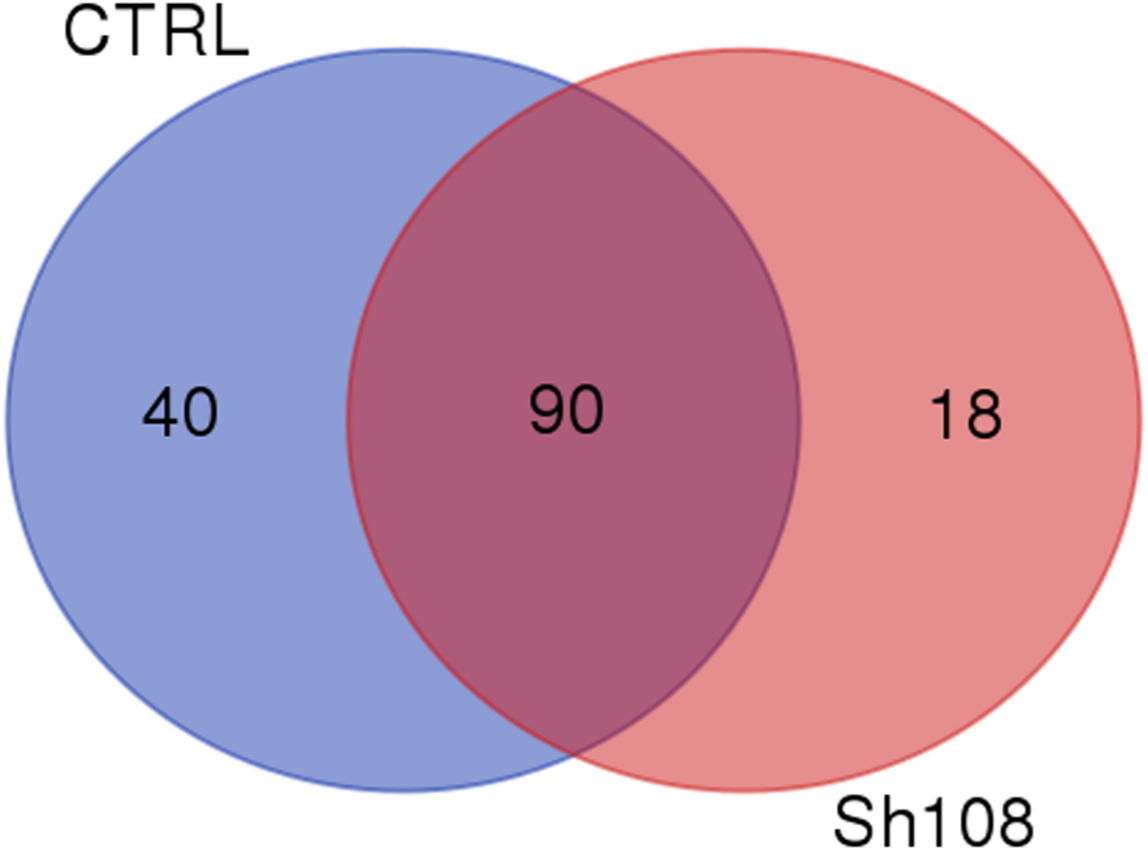
Intestinal core microbiota. Venn diagram representing unique and shared OTUs between fish of the CTRL and Sh108 dietary groups.

### QIIME data analysis and taxonomic characterization of gut microbiome

The twelve fecal samples were processed via Illumina MiSeq platform and analysed using the QIIME pipeline. During bioinformatics analysis process, two CTRL samples were discarded following OTU-picking step, due to their inadequate number of sequences. The total number of reads taxonomically classified according to the Greengenes database was 394,611, which corresponded to an average number of 39,461 ± 13,626 reads per sample (Table 5). Sequences of eukaryotic origin were 51% of total reads. Sequencing data were exported as individual fastq files and deposited in the European Nucleotide Archive (EBI ENA) under the accession code: PRJEB25441.

**Table 5:** Alpha diversity results of gut microbiota of seabream fed two tested diets. Number of reads per sample assigned to OTUs, and alpha diversity metrics values (normalized at the lowest sample size: 20,052 reads) of gut microbial community of gilthead sea bream fed CTRL (*n* = 4) or Sh108 (*n* = 6) diets for 90 days. Data are expressed as means ± SD. Different letters indicate statistically significant differences between groups (Student’s *t*-test, *P* < 0.05).

Diet	Reads	Observed species	Good’s coverage	PD Whole tree	Chao1	Shannon
CTRL	26,828 ± 7,248^b^	160 ± 19	0.99 ± 0.0	13.8 ± 1.0	172 ± 19	3.3 ± 0.7
Sh108	47,883 ± 9,482^a^	154 ± 24	0.99 ± 0.0	13.6 ± 1.9	172 ± 21	2.4 ± 0.7
**Total number of reads taxonomically classified**					394,611	
**Mean number of reads/sample**					39,461 ± 13,626	
**Total number of OTUs**					259	

**Figure 3:**
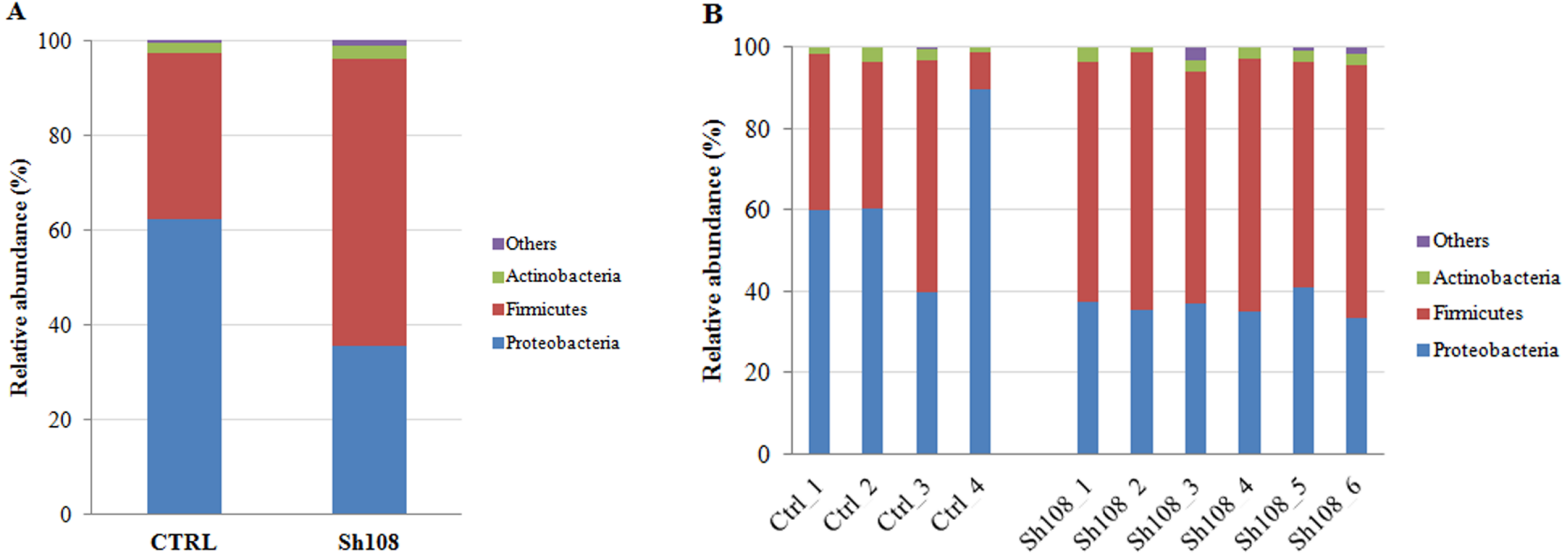
Relative abundance (%) of the overall most prevalent bacterial phyla in the gut of (A) all, and (B) individual fish fed with CTRL and Sh108 diets. ****All bacteria with an overall abundance of ≥ 1% were reported. Bacteria with lower abundance were pooled and indicated as “Others”.

**Figure 4:**
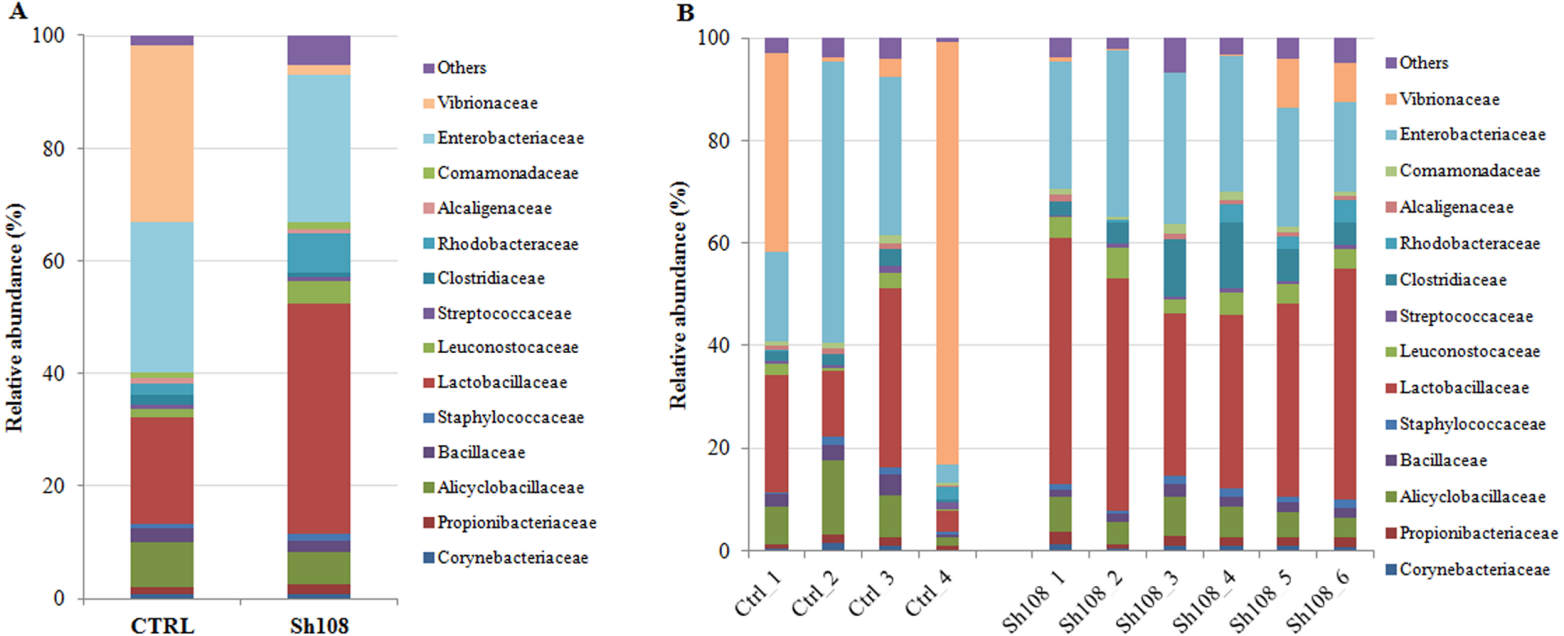
Relative abundance (%) of the overall most prevalent bacterial families in the gut of (A) all, and (B) individual fish fed with CTRL and Sh108 diets. All bacteria with an overall abundance of ≥ 0.5% were reported. Bacteria with lower abundance were pooled and indicated as “Others”.

**Figure 5:**
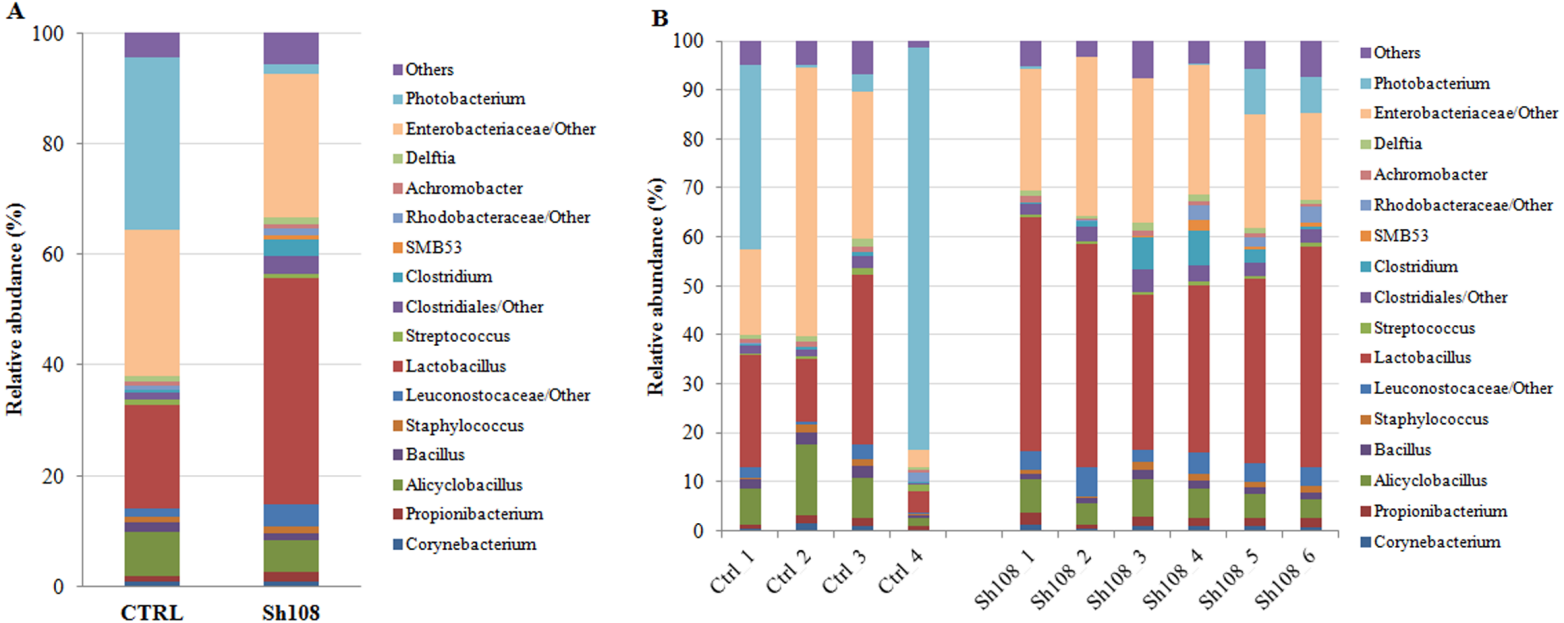
Relative abundance (%) of the overall most prevalent bacterial genera in the gut of (A) all, and (B) individual fish fed with CTRL and Sh108 diets. All bacteria with an overall abundance of ≥ 0.5% were reported. Bacteria with lower abundance were pooled and indicated as “Others”.

We identified 259 OTUs at 97% identity in sea bream fecal samples (Dataset S2). Ninety OTUs constituted the core gut microbiota, i.e., those OTUs found in at least three out of the four control samples and at least five out of the six Sh108 samples (or OTUs present in at least 75% of fecal samples) and shared, regardless of the diet (Fig. 2). Among these, 43 OTUs were common to 100% of samples, showing a dominance of *Firmicutes* (26 OTUs) (Dataset S3). Good’s coverage values for both dietary groups were >0.99, indicating that sequencing coverage was attained and that the OTUs found in the samples were representative of the whole population (Table 5). The whole microbial community profile of samples, excluding reads from eukaryotic origin, was successfully outlined, resulting in nine phyla, 14 classes, 25 orders, 44 families, 75 genera, and 38 species (Dataset S2). However, only taxa with an overall abundance of more than 1% (at the phylum, class, and order level) and more than 0.5% (at family and genus level) were considered for statistical analysis. The mean relative abundance changes at species level between groups were not considered to be informative since the number of unassigned sequences was remarkable (74-92%) and they were consequently excluded from analysis. Therefore, considering only the most abundant taxa, the overall gut microbial community was comprised of three phyla, six classes, eight orders, 14 families, 12 genera, and 13 species. The profiles of intestinal microbial communities for each dietary group and individual fish are presented at the phylum (Figs. 3A, 3B), family (Figs. 4A, 4B), and genus (Figs. 5A, 5B) level.

**Table 6:** Mean relative abundance (%) ± SD of the most prevalent bacterial phyla, classes, orders, families, and genera found in fecal samples of gilthead sea bream fed with two tested diets.

	CTRL	Sh108	*P*-value	Benjamini Hochberg *P*-value
**Phylum**				
*Actinobacteria*	2.14 ± 1.09	2.68 ± 0.78	0.413	0.591
*Firmicutes*	35.11 ± 19.63	60.64 ± 1.63	0.021	0.135
*Proteobacteria*	62.38 ± 20.50	35.60 ± 1.63	0.022	0.135
**Class**				
Actinobacteria	2.16 ± 1.10	2.80 ± 0.89	0.367	0.591
Bacilli	33.01 ± 18.52	55.25 ± 6.51	0.039	0.209
*Clostridia*	2.47 ± 1.54	7.60 ± 4.67	0.069	0.211
*Alphaproteobacteria*	1.11 ± 0.92	2.21 ± 2.11	0.339	0.591
*Betaproteobacteria*	2.07 ± 0.85	2.53 ± 1.18	0.531	0.671
*Gammaproteobacteria*	58.63 ± 20.98	28.41 ± 3.01	0.014	0.135
**Order**				
*Actinomycetales*	2.16 ± 1.10	2.80 ± 0.89	0.367	0.591
*Bacillales*	11.80 ± 7.18	9.52 ± 2.00	0.513	0.668
*Lactobacillales*	21.21 ± 14.52	45.73 ± 8.07	0.014	0.135
*Clostridiales*	2.47 ± 1.54	7.60 ± 4.67	0.069	0.211
*Rhodobacterales*^a^	0.64 ± 1.18	1.73 ± 2.13	0.241	0.545
*Burkholderiales*	1.95 ± 0.77	2.12 ± 0.96	0.792	0.874
*Enterobacteriales*	26.72 ± 21.86	26.19 ± 5.56	0.959	0.959
*Vibrionales*^a^	31.53 ± 38.20	1.78 ± 3.22	0.066	0.211
**Family**				
*Corynebacteriaceae*	0.75 ± 0.58	0.83 ± 0.34	0.820	0.874
*Propionibacteriaceae*	1.15 ± 0.44	1.75 ± 0.59	0.138	0.364
*Alicyclobacillaceae*	7.98 ± 5.33	5.70 ± 1.66	0.389	0.591
*Bacillaceae*	2.5 ± 1.42	1.88 ± 0.33	0.363	0.591
*Staphylococcaceae*	0.88 ± 0.67	1.23 ± 0.48	0.402	0.591
*Lactobacillaceae*	18.75 ± 13.23	40.90 ± 7.41	0.015	0.135
*Leuconostocaceae*	1.55 ± 1.34	4.15 ± 1.21	0.018	0.135
*Streptococcaceae*	0.89 ± 0.55	0.65 ± 0.18	0.446	0.599
*Clostridiaceae*	1.94 ± 1.16	7.02 ± 4.63	0.068	0.211
*Rhodobacteraceae*^a^	0.64 ± 1.18	1.73 ± 2.13	0.241	0.545
*Alcaligenaceae*	0.90 ± 0.32	0.85 ± 0.43	0.834	0.874
*Comamonadaceae*	1.00 ± 0.43	1.14 ± 0.53	0.689	0.822
*Enterobacteriaceae*	26.72 ± 21.86	26.18 ± 5.55	0.958	0.959
*Vibrionaceae*^a^	31.29 ± 38.13	1.75 ± 3.20	0.066	0.211
**Genus**				
*Corynebacterium*	0.75 ± 0.58	0.83 ± 0.34	0.820	0.874
*Propionibacterium*	1.15 ± 0.44	1.75 ± 0.59	0.138	0.364
*Alicyclobacillus*	7.98 ± 5.33	5.70 ± 1.66	0.389	0.591
*Bacillus*	1.78 ± 0.89	1.34 ± 0.31	0.333	0.591
*Staphylococcus*	0.86 ± 0.65	1.18 ± 0.51	0.439	0.599
*Lactobacillus*	18.73 ± 13.20	40.86 ± 7.36	0.014	0.135
*Streptococcus*	0.89 ± 0.55	0.62 ± 0.15	0.400	0.591
*Clostridium*	0.39 ± 0.24	3.09 ± 3.33	0.144	0.364
*SMB53*	0.11 ± 0.08	0.70 ± 0.93	0.258	0.554
*Achromobacter*	0.83 ± 0.31	0.77 ± 0.38	0.802	0.874
*Delftia*	0.95 ± 0.38	1.11 ± 0.52	0.629	0.822
*Photobacterium*^a^	31.04 ± 38.02	1.74 ± 3.20	0.066	0.211

**Notes.**

Significance of the differences (*P* < 0.05) was obtained by Student’s *t*-test or non-parametric Mann-Whitney *U* test (a) depending on normal distribution of data. Benjamini-Hochberg FDR method was applied for multiple test correction with Q set to 0.20.

Different *α*-diversity metrics were applied, including observed species count, phylogenetic diversity (PD Whole tree), and Chao1 and Shannon indices. All the rarefaction curves, normalized to the sample with the lowest number of sequences (20,052 reads), tended to plateau (Figs. S1A–S1C). As reported in Table 5, neither of the indices of diversity and species richness was affected by adding of SILOhealth 108Z to the diet. In particular, Shannon diversity index reached a stable value in all samples, indicating that bacterial diversity in these communities was mostly covered and did not differ between the two experimental groups. Only the number of reads was significantly higher in Sh108 samples compared to control.

### Analysis of intestinal microbiome changes in response to different diets

To understand the between-group differences, the mean relative abundances of individual taxa were compared and the results are reported in Table 6. *Firmicutes*, *Proteobacteria* and *Actinobacteria* represented the dominant phyla in both experimental groups (Fig. 3A). Among them, amount of *Firmicutes* and *Proteobacteria* were significantly influenced by dietary monoglycerides supplementation. Our data revealed that the relative abundance of *Firmicutes* was significantly higher (60.64 ± 1.63%) in fish fed with diet Sh108 than in fish fed the control diet (35.11 ± 19.63%) (Table 6). In contrast, fish fed the control diet were characterized by a higher percentage of bacteria assigned to *Proteobacteria* phylum (62.38 ± 20.50%) than fish receiving diet Sh108 (35.60 ± 1.63%) (Table 6). *Bacilli* and *Gammaproteobacteria* classes were dominant in both dietary groups. However, fewer *Gammaproteobacteria* were found in the group Sh108 (28.41 ± 3.01%) than in the control group (58.63 ± 20.88%) (Table 6). In the same fish, at order level, a higher percentage of *Lactobacillales* was found. The increased proportion of *Lactobacillales* was due to a significant enrichment in bacteria belonging to *Lactobacillaceae* (40.90 ±7.41%) and *Leuconostocaceae* (4.15 ± 1.21%) families in comparison to the control group (Fig. 4A, Table 6). Accordingly, the number of bacteria assigned to the *Lactobacillus* genus was significantly higher in Sh108 samples (Fig. 5A, Table 6). At the species level, the number of unassigned bacteria was sizeable, more than 90% for Sh108 group and around 70% for control, thus making a comparison between the two groups meaningless at this taxonomical level. However, although the percentage of unassigned sequences was remarkable at this taxonomical level, the only species of *Lactobacillus* identified, namely *L. agilis,* was found at a higher percentage in fish receiving Sh108 diet than in control group (0.15%).

### Beta diversity metrics of gut bacterial communities

**Figure 6:**
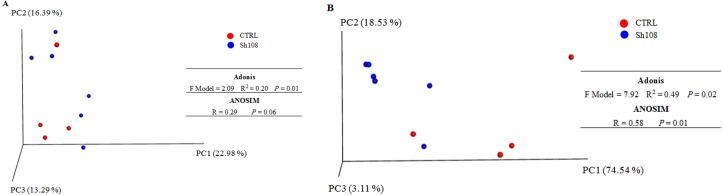
Beta diversity metrics. Principal Coordinate Analysis of (A) Unweighted, and (B) Weighted Unifrac distances of gut microbial communities associated to two experimental diets. Each dot represents an individual sample plots according to its microbial profile at genus level. Results of Permutational multivariate analysis of variance (adonis function) and Analysis of similarity (ANOSIM) are reported next to the PCoA plot to which they are referred. Significance was set at *P* < 0.05.

QIIME pipeline ‘beta_diversity_trough_plots.py’ was used to compute microbial beta diversity metrics; both weighted and unweighted UniFrac analyses were performed. Sample UniFrac distances were visualized using principal coordinate analysis (PCoA) onto a three-dimensional plot (Figs. 6A, 6B). Unweighted PCoA showed no sharp separation between samples, which clustered together regardless of the diet (Fig. 6A). Contrariwise, weighted PCoA revealed a clear clustering of samples by diet and principal coordinates PC1 and PC2 together explained 93% of the variation between individuals (Fig. 6B). The permutational multivariate analysis Adonis totally confirmed the PCoA plots results, revealing a significant difference in microbial communities of gut microbiota between the two groups (F Model = 7.92, *P* = 0.02; *R*^2^ = 0.49). The *R*^2^ value, from Adonis test, indicated that the sample grouping explained the 49% of the variation in distances. Similarly, ANOSIM test was significant only for weighted Unifrac distance matrix (*P* = 0.01; *R* = 0.58), indicating that the divergences between samples were due more to differences in bacterial abundance rather than to the presence or absence of specific taxa. Results of multivariate analysis are summarized in Fig. 6.

## Discussion

We tested a specific mix of 1-monoglycerides of short- and medium-chain organic acids (SILOhealth 108Z) in the diet of gilthead sea bream, to determine the effects on fish intestinal microbiota and growth performance. This product is a synergic combination of short and medium chain 1-monoglycerides (from C3 to C12), particularly rich in monobutyrin. It has been widely demonstrated that butyrate, despite being the least abundant of the three-primary gastrointestinal SCFAs (acetate, propionate and butyrate), exerts important protective and anti-inflammatory functions in the gut of several fish species, ultimately enhancing gut health and improving fish performance (Benedito-Palos et al., 2016; Liu et al., 2014; Terova et al., 2016; Rimoldi et al., 2016). These previous, promising results prompted the idea that, as feed additive, butyric acid monoglycerides, could represent an effective strategy to improve fish growth performance, feed conversion, and disease resistance by promoting the establishment of a healthy intestinal microbiota. Indeed, esterification with glycerol protect butyric acid from being absorbed in the upper part of the digestive system targeting its release in the deeper tracts of intestine where butyrate would exert its major functions.

Use of monoglycerides as feed additive has been widely investigated in poultry (Bedford & Gong, 2018; Yang et al., 2018; Jahanian & Golshadi, 2015; Leeson et al., 2005). On the contrary, research dealing with their use in aquaculture is very scarce to date, despite the increasing commercial interest in the use of SCFAs and MCFAs in aquafeeds for farmed fish species. In this perspective, our findings represent a first contribution which could help to fill this knowledge gap.

We tested a dietary inclusion level of 0.5% for SILOhealth 108Z. This inclusion level was chosen based on studies conducted in Pacific white shrimp (*Penaeus vannamei*) and white sturgeon (*Acipenser transmontanus*) that were recently presented at some aquaculture conferences by Parini & Paoli (2016), and Parini (2016). The authors of these studies reported that the inclusion of 0.5% of SILOhealth 108Z in shrimp feed increased SGR and improved FCR, whereas in sturgeon infected with *Aeromonas hydrophila*, the addition of 0.8% of SILOhealth 108Z to the diet, improved fish growth performance, and increased the survival rate. However, considering that no bacterial challenge was planned in our study for gilthead sea bream, a nutritional dosage of 0.5% of SILOhealth 108Z was decided to be included in the diet of this species.

The dietary supplementation of 0.5% SILOhealth 108Z did not significantly improve fish growth performance. However, even if not significant, SGR mean value of fish receiving Sh108 diet showed an improvement of 3% in comparison to control fish. Interestingly, even if the biological FCR did not differ between two groups, the economic FCR value was lower (improved) in fish fed with Sh108 diet. The eFCR is a very strong tool for farmers and feed companies to monitor the performance of feeds as it takes into account not only the nutritional value of the feed, but also the health status of the fish (Robb & Crampton, 2013). Indeed, factors well outside the control of the feed quality, such as fish disease and mortalities, can strongly affect eFCR and in order to reduce (improve) the eFCR, farmers should follow a series of corrective actions as described in Robb & Crampton (2013).

Similarly to the present study, no consistent effects in growth rates were observed in rainbow trout (Gao et al., 2011), European sea bass (Terova et al., 2016; Rimoldi et al., 2016) or gilthead sea bream fed dietary butyrate (Benedito-Palos et al., 2016). On the other hand, a diet supplemented with medium-chain fatty acids in the form of a sodium salt of coconut fatty acid distillate enhanced the overall feed intake and growth rates of sea bream (Simó-Mirabet et al., 2017). As suggested by Ng & Koh (2011), in addition to the amount of organic acid included in the diet, various factors may influence fish growth, including organic acid type, fish species and age, diet composition, and farming condition, which could explain these apparently conflicting and inconsistent results reported in literature.

A precious contribution to our understanding of the controversial mechanism of action of organic acids could come from studies of fish gut microbiota. Recently, the advent of next-generation sequencing (NGS) technologies has substantially improved our knowledge of changes in the gut microbial ecosystem in fish, in response to a variety of factors, including diet. To the best of our knowledge, this study represents the first investigation on the effects of dietary 1-monoglycerides on gut bacterial community of gilthead sea bream. In agreement with previous metagenomics studies conducted on the same fish species, our results indicated that *Firmicutes* and *Proteobacteria* were the most dominant phyla of the gut microbiome regardless of the diet (Parma et al., 2016; Estruch et al., 2015). Similarly, Piazzon et al. (2017) found a dominance of *Proteobacteria* in intestine of juvenile sea bream unrelated to the diet; however, compared to our findings, the relative abundance of *Firmicutes* was much lower, from 0.5% to 27.9%. This divergence could be related to the fact that Piazzon and colleagues (2017) investigated only changes in the autochthonous bacterial community, whereas we considered both the luminal- (allochthonous) and mucosa-associated communities (autochthonous). Actually, *Firmicutes* are generally the dominant phylum of transient microbial community in the distal intestine with a relative abundance of around 70% (Parma et al., 2016; Estruch et al., 2015).

Although we did not observe an overall effect of 0.5% SILOhealth dietary supplementation on the bacterial richness and diversity, the composition of gut microbiota in terms of relative abundance of specific taxa, was significantly influenced by the dietary treatment. As revealed by weighted UniFrac PCoA of bacterial communities, there was a significant relationship between diet type and microbiota associated to fish intestine. Weighted UniFrac *β*-diversity measurement showed a clear clustering of samples by diet, statistically validated by ANOSIM and adonis test. Our data revealed that including SILOhealth 108Z in the diet was associated with a higher *Firmicutes*:*Proteobacteria* ratio than in the control diet, which instead favoured, the presence of *Proteobacteria*. Specifically, adding 1-monoglycerides to the diet induced a twofold increase in intestinal *Firmicutes* relative abundance as compared to the control diet. A similar trend was described in sea bream following butyrate dietary administration (Piazzon et al., 2017), but in this case a 139-fold increase with respect to the control diet was registered. The *Firmicutes* phylum includes different genera of lactic acid bacteria such as *Streptococcus*, *Lactobacillus*, and *Leuconostoc*. They are generally thought to be beneficial microorganisms associated with a healthy intestinal epithelium and are often used as probiotics for fish and other vertebrates; therefore, an increase in their number is mostly considered desirable (Kim, Bhatnagar & Kang, 2012; Askarian et al., 2011; Ringø& Gatesoupe, 1998). Moreover, *Firmicutes* include several bacterial genera, which play an important role in degrading otherwise indigestible carbohydrates, such as resistant starch and dietary fiber, thus contributing to a more efficient food energy utilization. In particular, the relative abundance of lactic acid bacteria belonging to the *Leuconostocaceae* and *Lactobacillaceae* families, the latter mainly represented by *Lactobacillus* genus, were positively affected by our tested feed additive. In agreement with our findings, dietary Na-butyrate supplementation increased the abundance of *Lactobacillus* and decreased the number of harmful bacteria *Aeromonas* and *Escherichia coli* in the intestine of grass carp (*Ctenopharyngodon idella*) (Tian et al., 2017). Similarly, the lactic acid bacteria, but not the total intestinal bacterial count, significantly increased in common carp fry fed different levels of a blend of SCFAs (Hoseinifar, Sun & Caipang, 2017). Furthermore, it has been reported that the supplementation of potassium diformate to plant protein-based diets stimulated the colonization of some lactic acid bacteria in the gut of tilapia (*Oreochromis niloticus*) (Abu Elala & Ragaa, 2015) and hybrid tilapia (*Oreochromis niloticus* ♀ × *Oreochromis aureus* ♂) (Zhou et al., 2009), whereas butyrate supplementation at 0.4% in a plant-based diet, induced a partial reversion to gut microbial phenotype of fish fed control diet (based on fishmeal and fish oil), with a decrease in *Photobacterium* (Piazzon et al., 2017). A similar effect was found in our samples; indeed, two fish of the control group showed very high percentage of this bacterial genus, whereas the relative abundance of *Photobacterium* was definitely less in all samples of Sh108 group. Actually, besides *Firmicutes,* the number of *Proteobacteria*, in particular *Gammaproteobacteria,* was affected by adding SILOhealth 108Z to the diet. Indeed, sea bream fed with Sh108 diet showed a reduced percentage of this taxon in comparison to control group. The dominance of *Proteobacteria* phylum in gut microbiome has been described in several marine carnivorous fish (Sullam et al., 2012), including gilthead sea bream (Kormas et al., 2014; Piazzon et al., 2017; Estruch et al., 2015). However, the most abundant *Proteobacteria* harboured in the gut of sea bream from either a wild population or fed conventional fishmeal-based diets, are usually *Betaproteobacteria* (Desai et al., 2012) and not *Gammaproteobacteria*, as in the present study. Generally, a high amount of *Gammaproteobacteria* has been associated with vegetable ingredients in the diet (Piazzon et al., 2017; Desai et al., 2012; Estruch et al., 2015). Indeed, the *Gammaproteobacteria* class includes several species of bacteria, belonging, for example, to *Photobacterium* genus*,* capable to degrade cellulose. However, the *Proteobacteria* phylum includes also many potential pathogenic genera, such as *Pseudomonas*, the same *Photobacterium*, and *Vibrio*. Therefore, when this phylum represents the dominant clade of intestinal microflora, it might indicate an alteration in the gut microbiota balance. An imbalanced microbiota, could negatively affect the intestinal immune mechanisms, thus contributing to easier development of diseases in fish (Savas, Kubilay & Basmaz, 2005). In the present study, 0.5% of organic acid monoglycerides in the diet was sufficient to significantly reduce the amount of *Proteobacteria* in the intestine of gilthead sea bream and, at the same time, to favour the proliferation of *Firmicutes*. Interestingly, Kollanoor and colleagues (2007) demonstrated *in vitro* antibacterial activity of caprylic acid (C9) and its monoglyceride that is a component of SILOhealth 108Z blend, against fish pathogens, including *Edwardsiella* species that belong to *Gammaproteobacteria* class. Additionally, low concentrations of SILOhealth 108Z (from 0.01% to 0.1%) inhibited growth of pathogenic bacteria *in vitro*, without inhibiting the beneficial *Lactobacillus plantarum* and *Lactobacillus acidophilus* (Parini & Paoli, 2016). This *in vitro* test proved that SILOhealth 108Z selectively exerts antibacterial action against *Vibrio parahaemolyticus*, *Vibrio mimicus*, *Aeromonas salmonicida*, *Aeromonas hydrophila*, *Bacillus cereus*, and *Photobacterium damselae*. Accordingly, the inclusion of SILOhealth 108Z in white sturgeon, rohu (*Labeo rohita*) and shrimp diets reduced the mortality caused by pathogenic bacteria *A. hydrophila* and *V. parahaemolyticus* (Parini, 2016). The antimicrobial action of SILOhealth 108Z is strictly related to the amphipathic structure of monoglycerides that enables them to interact with cell membranes of several enteric pathogenic bacteria, thus altering membrane integrity and causing inhibition of bacterial growth up to cell death (Yoon et al., 2018; Salsali, Parker & Sattar, 2008).

In this regard, even *Lactobacilli* could have an active role in host defense against pathogenic bacterial invasion at the intestinal level. It is known that lactic acid bacteria inhibit the growth of pathogens by producing antibacterial compounds, such as lactic acid, hydrogen peroxide, and bacteriocins and by releasing biosurfactants. These are a structurally diverse group of surface-active compounds synthesized by microorganisms and characterized by amphipathic nature. Biosurfactants enhance the solubility of water-insoluble compounds, facilitating their uptake into the cell. They participate in processes such as biofilm formation and defense against other microorganisms by affecting microorganisms’ adhesion to different surfaces and exhibiting antibacterial activity. In our study, *L. agilis* was the only species of *Lactobacillus* present in small amounts in fish fed Sh108 diet, but not in fish fed the control diet. Also of interest, it has been recently reported that this bacterial species has the ability to produce a biosurfactant compound, which is a glycoprotein with antimicrobial and anti-adhesive activities that are effective against pathogens such as *Staphylococcus aureus*, *Streptococcus agalactiae* and *Pseudomonas aeruginosa* (Gudiña et al., 2015).

## Conclusions

In summary, the present study indicated that there were no differences in growth performance between gilthead sea bream fed the diet supplemented with 0.5% of SILOhealth 108Z and fish fed the control diet. Economic feed conversion ratio (eFCR) was, instead, significantly improved by dietary administration of 1-monoglycerides. Our findings clearly indicated that SILOhealth 108Z positively modulated the fish intestinal microbiota by increasing the relative abundance of beneficial lactic acid bacteria, namely, *Lactobacillus*. Therefore, the specific composition of 1-monoglycerides of short- and medium-chain fatty acid contained in SILOhealth 108Z has great potential as a feed additive in aquaculture. The present study provides a further confirmation that it possible through diet manipulation to obtain positive effects on gut microbiota, which is known to have a very important role in growth performance, feed conversion, and disease resistance of farmed fish. However, further experiments are needed to elucidate which feed ingredients have the highest impact on changes in the gut microbiota and how these changes can interact with host metabolism.

##  Supplemental Information

10.7717/peerj.5355/supp-1 Fig. S1 Alpha diversity metrics Rarefaction curves of fecal microbial communities from sea bream fed two tested diets normalized at the lowest sample size (20,052 reads). (A) Observed species, (B) species richness (Chao1), (C) PD whole tree. Data points represent the mean values.

10.7717/peerj.5355/supp-2 Dataset S1 List of identified taxa in the feeds

10.7717/peerj.5355/supp-3 Dataset S2 List of identified OTUs in the fecal samples from each fish

10.7717/peerj.5355/supp-4 Dataset S3 List of identified core OTUs

10.7717/peerj.5355/supp-5 Dataset S4 Raw data for fish growth

## Additional Information and Declarations

### DNA Deposition

The following information was supplied regarding the deposition of DNA sequences: Sequencing data has been deposited in European Nucleotide Archive (EBI ENA) under the accession code: PRJEB25441.

### Data Availability

The following information was supplied regarding data availability: All the raw data are included in the Tables, Supplemental Materials and Figshare: Terova, Genciana, and 0000-0002-1995-263x. “Sea Bream Gut Bacterial DNA Sequencing”. 25 July 2018. Web. 26 July 2018. DOI: 10.6084/m9.figshare.5858310.v1.
