# Supplementary figures and images for: Effect of a specific composition of short- and medium-chain fatty acid 1-Monoglycerides on growth performances and gut microbiota of gilthead sea bream (Sparus aurata)
Source: PeerJ. 2018 Jul 31;6:e5355. doi: 10.7717/peerj.5355 (PMC6074759; doi:10.7717/peerj.5355)

**A**

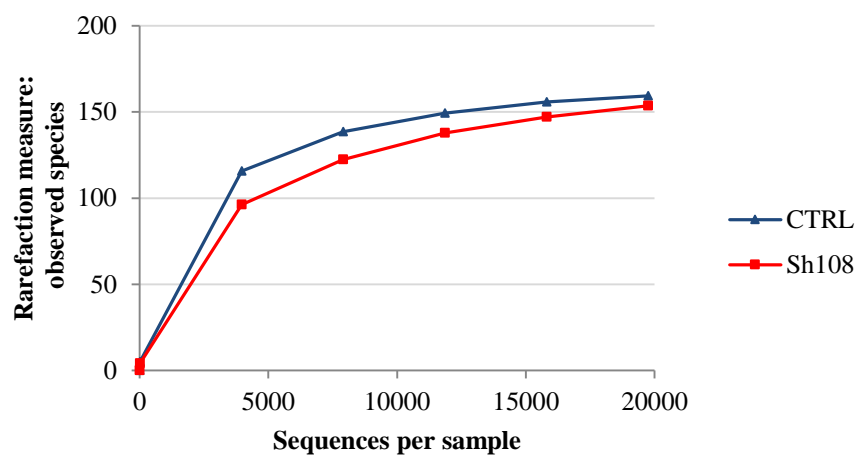

**B**

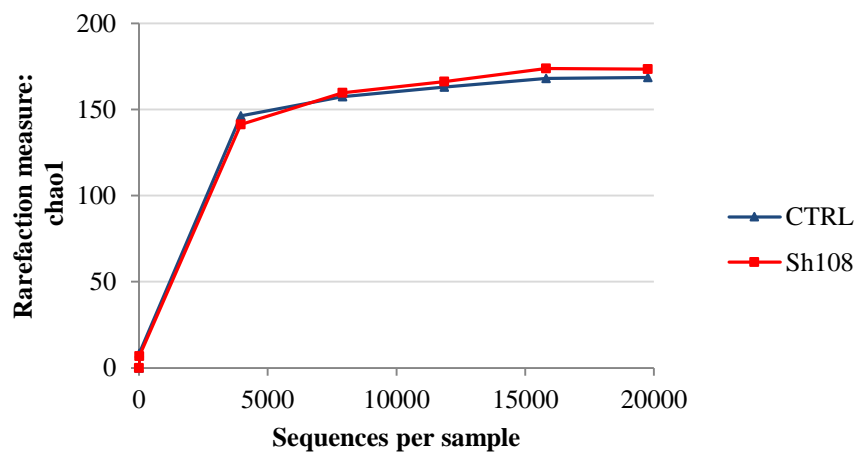

**C**

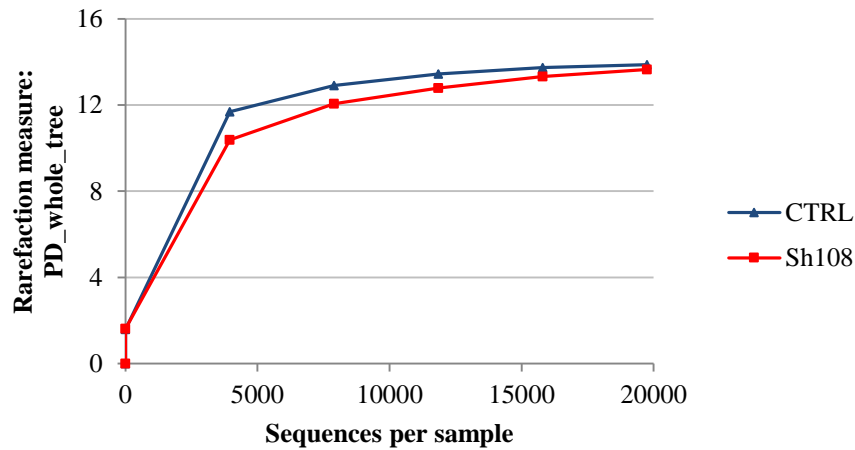

Supplement: Fig. S1 — Rarefaction curves of fecal microbial communities from sea bream fed two tested diets normalized at the lowest sample size (20,052 reads). (A) Observed species, (B) species richness (Chao1), (C) PD whole tree. Data points represent the mean values. [file peerj-06-5355-s001.pdf]
